# Comparison of cytokine profiles in the aqueous humor of eyes with pseudoexfoliation syndrome and glaucoma

**DOI:** 10.1371/journal.pone.0182571

**Published:** 2017-08-10

**Authors:** Justus G. Garweg, Souska Zandi, Isabel B. Pfister, Magdalena Skowronska, Christin Gerhardt

**Affiliations:** 1 Swiss Eye Institute and Berner Augenklinik am Lindenhofspital, Bern, Switzerland; 2 University of Bern, Bern, Switzerland; University of Tennessee Health Science Center, UNITED STATES

## Abstract

**Purpose:**

To compare the cyto- and chemokine profiles in the aqueous humor of PEXS eyes in the absence or presence of secondary glaucoma with or without luxation of the intraocular lens (IOL).

**Methods:**

Samples of aqueous humor were collected intraoperatively from 20 healthy controls and from 73 eyes with PEX-syndrome, which was manifested in the absence of any other local or systemic desease. The latter group was sub-devided into eyes with an early form of PEX-syndrome in the absence of complications (PEX, *n* = 33), those with a late form of PEX-syndrome and glaucoma (PEXG, *n* = 30), and those with a late form of PEX-syndrome with luxation of the IOL that required surgery (PEXL, *n* = 10). The samples were analyzed in parallel after storage at -80°C. The levels of 40 cytokines were simultaneously quantified using the Bio-Plex® multiplex beads system. The inter-group data were statistically compared using the Kruskal-Wallis test (*p* ≤ 0.01).

**Results:**

PEX and PEXG were comparable in their cytokine profiles for all 40 cytokines, whereas the cytokine profile in PEXL-eyes revealed higher levels of all but 5 cytokines (CXCL13, CCL27, IL-2, CCL3, CCL20; *p* ≤ 0.01). This latter finding is indicative of a non-specific inflammatory reaction in the context of IOL-luxation. The concentrations of 6 cytokines lay below the detection limit in all groups.

**Conclusions:**

The local up-regulation of 85% of the detectable cytokines in the aqueous humor of PEXL-eyes may be linked either with a progression of the disease or a breakdown of the antero-posterior barrier in the context of IOL-luxation.

## Introduction

Pseudoexfoliation syndrome (PEXS) is a clinically late-manifesting generalized fibrillopathy, which is characterized by a pathological accumulation of abnormal extracellular material in intra- and extraocular tissues. Although many factors are known to influence the development of the PEX-syndrome the exact aetiology of the disease remains elusive [1]. Changes in specific genes such as polymorphism of that for the enzyme lysyl oxidase-like 1 (LOXL1), have been demonstrated in almost all of the affected individuals, irrespective of their genetic background [2]. LOXL1 plays a pivotal role in the metabolism of the extracellular matrix, and appears to be specifically involved in the formation and stabilization of elastic fibres [3], as well as in the intracellular linkage of collagen and elastin. Two single nucleotide polymorphisms in the LOXL1-gene have been associated with the accumulation of amyloid-like protein fibres in intra- and extraocular tissues and, on this basis, with the pathophysiology of the disease [2,4].

Age, race and gender, as well as free radicals and oxidative damage, contribute to the development of the PEX-syndrome [5,6].

The deposition of PEX-material along the inner surfaces of the anterior segment is associated with progressive atrophy of the iris, weakness of the zonular fibres and an increase in the outflow resistance of the trabecular meshwork. In the long term, these changes almost inevitably lead to luxations of the natural or an implanted lens, a rise in intraocular pressure and the development of secondary glaucoma [7].

Clinically, PEX-syndrome usually begins unilaterally. In 24–52% of the cases, the fellow eye is also implicated at a later stage [8]. In the seemingly monocular cases, changes in the dynamic of the aqueous humor, notably pigmentation of the trabecular meshwork and an elevation in intraocular pressure, have, however, been reported in the “unaffected” partner eyes [8,9].

Secondary glaucoma does not necessarily develop in association with the PEX-syndrome. However, in 25% of all cases of glaucoma, PEXS is manifested^10^. Pathophysiologically, an elevation of the intraocular pressure appears to be the result of a local production and deposition of PEX-material by cells of the trabecular meshwork and by those of Schlemm's canal resulting in degenerative changes in Schlemm's canal and in the juxtacanalicular tissues [10]. A reduced tolerance to intraocular pressure (IOP) elevation associated with degenerative changes in the lamina cribrosa leads to rapidly progressive nerve fibre damage [3,7]. Furthermore, the deposition of PEX-material in several extraocular locations has been linked with an increase in cardiovascular and cerebrovascular morbidity [11].

Pro-inflammatory cytokines have been implicated in the onset and the progression of various fibrotic disorders and in the pathophysiology of glaucoma, as well as of PEXS [12–15]. However, the roles that they play in the aetiology of these diseases have not as yet been fathomed.

In the present study, wecompared the concentrations of 40 cyto- and chemokines in the aqueous humor in eyes in which an early form of the PEX-syndrome was manifested in the absence of complications (PEX), of those with a late form of PEX-syndrome and glaucoma (PEXG), and of those with a late form of PEX-syndrome, glaucoma and luxation of the intraocular lens (IOL) that required surgery (PEXL). The purpose of this analysis was to ascertain whether the progression of the PEX-syndrome is associated with changes in the intraocular cytokine environment.

## Patients and methods

### Patients

This prospective study of a case-series of patients with PEX, PEXG and PEXL was approved by the local Institutional Ethics Board of the University of Bern, Switzerland (registration number: 152/08). It was undertaken with the informed written consent of each of the participants and was fully compliant with the tenets of the Declaration of Helsinki.

A total of 73 eyes with PEX-syndrome in as many patients, who were otherwise healthy, were enrolled. Twenty healthy eyes that were undergoing phacoemulsification surgery served as controls. The patients in the control group were not suffering from or being treated for any other systematic disease.

Individuals with a history of any intraocular disorder other than PEX-syndrome, including diabetes with or without diabetic retinopathy, and those who had undergone uncomplicated phacoemulsification up to 6 months prior to the time of enrollment, were excluded from the analysis.

### Collection of aqueous humor

Samples of aqueous humor were collected at the onset of phacoemulsification surgery. They were maintained at ambient temperature for up to 6 hours prior to storage at -20°C for maximally 3 months and thereafter (January 2014 to January 2016) at -80°C until the time of the analysis at the Berner Augenklinik am Lindenhof, Bern, Switzerland.

### Cytokine analyses

The cytokine analyses were performed using the Bio-Plex® multiplex beads system (Bio-Plex 100 array reader with Bio-Plex Manager software version 6.1, Bio-Rad, Hercules, California). With this system, it is possible to quantify in parallel the concentration of numerous cytokines and chemokines in a single sample with small volume. In the present study, the levels of 40 pro- and anti-inflammatory chemo- and cytokines were quantified in each sample of aqueous humor. To this end, fluorescently-labelled magnetic microspheres were coupled to specific capture antibodies and mixed with the samples in which the cytokine concentrations were unknown. Biotinylated detection antibodies and streptavidin R-phycoerythrin were introduced into the mixture, which was then analyzed by flow cytometry. Two lasers identified the microsphere type and quantified the amount of the bound antigen. The readings were compared with the values on a standard curve, which represented the average of triplicate standard dilutions of the corresponding cytokines that were run on the same plates. All measurements were performed in a blinded manner by a laboratory technician who was experienced in executing the technique.

### Statistical analysis

Numerical data are presented as mean values ± standard deviation (SD). The concentrations of several of the cytokines in some of the samples lay below the curve fit of the standards. To avoid the bias that would have been introduced by excluding these data, the concentrations of the implicated cytokines were set at half of the lower cut off of the test system, which was usually about 1 pg/ml. Outliers at the other end of the spectrum (higher than the mean ± 2 SD) were identified via boxplots and were excluded from the statistical analysis.

The Shapiro-Wilk test was implemented to ascertain whether the data were or were not normally distributed. Since the criteria for a normal distribution were not fulfilled, the non-parametric Kruskal-Wallis test was used for the inter-group comparisons, with the level for statistical significance being set at *p* ≤ 0.01. Total Protein Concentration, IgG and Albumin measurements were statistically evaluated using Student’s *t*-test, with the level for statistical significance being set at *p* < 0.05. Correlations between cytokine concentrations and patient`s age and IOP were calculated using Spearman`s correlation test. The statistical analysis was performed using SPSS (version 23.0; IBM SPSS Statistics, Armonk, NY, USA).

## Results

### Patients

Three groups of PEX patients were composed of individuals of comparable age and gender at the time of inclusion (Table 1). The healthy group differed in age from early (p = 0.013) and PEXL-group (p = 0.003), but not from the PEXG-group (p = 0.13). Neither the eyes of the healthy controls nor those that manifested PEXS at an early stage were being treated for glaucoma. In the PEXG-group, medication for glaucoma was being administered in 26 of the 30 cases (87%); the other four had undergone filtering surgery at an earlier time point. In the PEXL-group 6 of the 10 patients (60%) were undergoing glaucoma treatment (Table 1).

**Table 1:** Clinical data appertaining to the different groups.

	Controls	Early PEX	Late PEX	Late PEX+luxation
n-number	20	33	30	10
age *mean (range)*	68.32 *(28*.*9–87*.*7)*	81.19 *(65*.*6–97*.*8)*	79.30 *(66*.*3–90*.*43)*	84.24 *(68*.*7–92*.*4)*
gender	50% male, 50% female	39.4% male, 60.6% female	30% male, 70% female	20% male, 80% female
glaucoma medication	none	none	86.7%	60%

Regarding the correlation of age and IOP cytokines, no consistent or systematic correlations were identified. Nevertheless, we observed positive correlations for four cytokines (CXCL1, CXCL9, CCL15 and CCL25) in the healthy group and for three cytokines (IL-8, CCL2 and CXCL 16) in the early PEX group. In the PEXG-group we found an association of seven cytokines with age (CCL26, IL-16, IL-1 beta, CXCL11, CXCL9, CCL15 and TNF-alpha), whereas no significant correlation for any of the 40 cytokines for the PEXL group were detected (data shown in S1 Table). Regarding the cytokine correlation with IOP, solely in the PEXG-group a correlation for three cytokines was detected (CXCL13, CCL8, CCL3; S2 Table).

### Cytokine analyses

**Fig 1:**
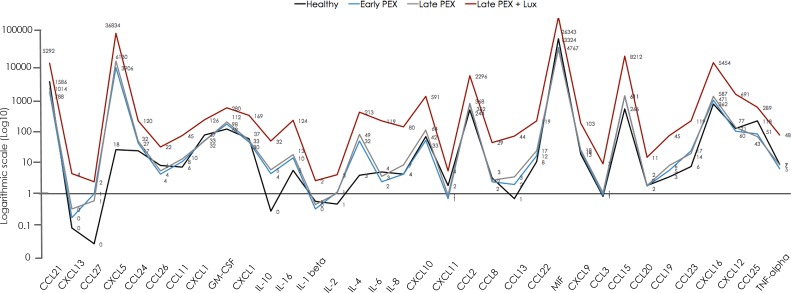
Cytokine profiles of 40 cytokines in the aqueous humor of healthy eyes, eyes with PEX, PEXG and PEXG with luxation, expressed on a logarithmic scale.

**Fig 2:**
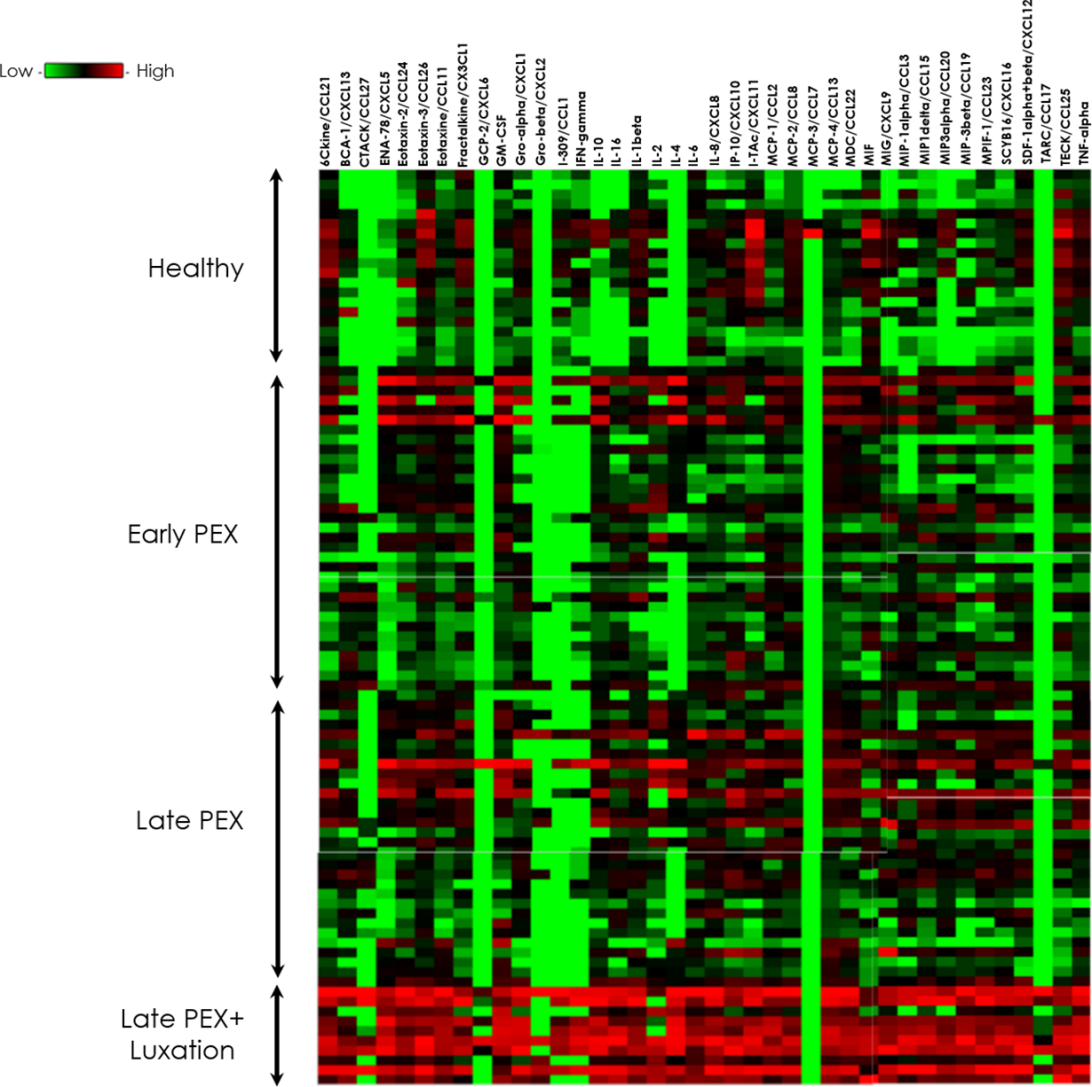
Visualization of the intergroup differences using a heat map chart for all 40 cytokines in early, late PEX and PEXG with IOL-luxation (*light green* indicates low levels, whereas *dark red* indicates elevated levels).

The cytokine concentrations (pg/ml) in the samples of aqueous humor are displayed numerically in Table 2, as cytokine profiles in Fig 1 and as heat-maps in Fig 2. The corresponding *p*-values are given in Table 3.

**Table 2:** Mean and standard deviation appertaining to the 34 detectable cytokines and chemokines whose levels in the aqueous humor were quantified using the Bio-Plex multiplex beads system.

Cytokine	Healthy		Early PEX		Late PEX		late PEX + luxation	
	Mean (pg/ml)	SD	Mean (pg/ml)	SD	Mean (pg/ml)	SD	Mean (pg/ml)	SD
CCL21	1585.9	1007.4	787.8	367.3	1013.5	469.0	5292.0	3522.7
CXCL13	0.1	0.1	0.2	0.2	0.4	0.3	3.7	3.8
CCL27	0.0	0.0	1.0	1.6	0.6	1.6	2.2	5.1
CXCL5	18.2	27.0	3905.5	4318.2	6130.1	6044.9	36834.2	18300.0
CCL24	16.8	6.7	27.2	12.2	32.4	16.8	120.2	70.3
CCL26	6.4	4.8	3.7	1.6	4.5	1.8	21.8	11.7
CCL11	5.7	2.3	8.2	4.2	9.8	5.0	45.1	18.7
CX3CL1	47.2	24.5	33.2	13.1	32.0	12.0	126.1	91.4
GM-CSF	69.0	20.8	98.0	33.1	111.7	71.5	280.3	107.2
CXCL1	36.7	17.1	29.8	17.1	32.8	18.7	168.8	93.6
IL-10	0.3	0.4	3.8	1.5	4.7	2.7	31.6	16.4
IL-16	4.7	5.6	10.4	5.9	13.1	9.4	123.6	91.5
IL-1 beta	0.6	0.4	0.4	0.3	0.5	0.4	2.3	1.4
IL-2	0.5	0.6	1.1	1.2	1.0	1.0	3.5	2.7
IL-4	3.4	0.9	32.0	31.7	48.6	40.6	212.5	73.3
IL-6	4.1	2.7	2.2	0.8	3.1	1.5	118.6	104.9
IL-8	3.7	2.1	3.7	1.4	6.5	4.1	79.8	66.4
CXCL10	42.0	32.1	33.4	23.2	64.2	61.1	590.8	609.7
CXCL11	1.7	1.3	0.7	0.4	0.9	0.5	4.4	2.4
CCL2	244.5	68.2	368.1	125.5	361.6	117.1	2296.5	1696.5
CCL8	2.8	2.1	2.2	1.1	2.4	1.8	28.9	30.6
CCL13	0.7	0.5	1.8	1.2	3.0	2.2	43.9	39.1
CCL22	8.2	3.9	12.1	4.3	16.8	8.9	118.8	90.6
MIF	26343.3	14414.9	14766.7	9515.2	13323.8	13160.4	108694.3	106911.8
CXCL9	13.5	15.3	16.1	10.9	17.8	11.8	102.6	63.8
CCL3	0.8	0.7	0.9	0.5	1.1	0.7	7.2	5.3
CCL15	266.5	144.2	611.1	448.6	611.5	373.2	8211.6	7528.7
CCL20	1.7	2.6	1.7	1.2	1.7	0.9	10.7	7.3
CCL19	3.1	3.3	4.6	2.6	6.4	5.5	44.9	27.1
CCL23	6.0	3.9	16.8	16.8	14.0	7.7	118.8	95.7
CXCL16	362.2	94.0	471.4	166.3	586.9	232.9	5453.5	4162.0
CXCL12	71.2	39.5	59.7	34.5	76.5	48.0	690.8	563.9
CCL25	118.2	74.5	51.1	27.1	42.6	25.0	289.2	179.3
TNF-alpha	6.8	3.5	5.1	1.8	6.6	3.2	47.8	26.9

**Table 3:** Information (*p*-values) appertaining to the 34 detectable cytokines and chemokines whose levels in the aqueous humor were quantified using the Bio-Plex multiplex beads system.

	Kruskal-Wallis H-Test	Kruskal-Wallis H-Test	Kruskal-Wallis H-Test	Kruskal-Wallis H-Test	Kruskal-Wallis H-Test	Kruskal-Wallis H-Test
Cytokine	Healthy	Healthy	Healthy	Early PEX	Early PEX	Late PEX
	Early PEX	Late PEX	Late PEX+ luxation	Late PEX	Late PEX+ luxation	Late PEX+ luxation
CCL21	*p* = 0.006	*p* = .382	*p* = .062	*p =* .723	*p =* .0005	*p =* .0005
CXCL13	*p* = .39	*p* = .008	*p* = .0005	*p =* .767	*p =* .005	*p =* .139
CCL27	*p* = .037	*p* = .816	*p* = .550	*p =* 1.0	*p =* 1.0	*p =* 1.0
CXCL5	*p* = .003	*p* = .0005	*p* = .0005	*p =* 1.0	*p =* .0005	*p =* .002
CCL24	*p* = .039	*p* = .004	*p* = .0005	*p =* 1.0	*p =* .001	*p =* .005
CCL26	*p* = .224	*p* = 1.0	*p* = .005	*p* = 1.0	*p =* .0005	*p =* .0005
CCL11	*p* = .787	*p* = .080	*p* = .0005	*p = 1*.*0*	*p =* .0005	*p =* .*003*
CX3CL1	*p* = .610	*p* = .343	*p* = .532	*p* = 1.0	*p* = .012	*p* = .006
GM-CSF	*p* = .063	*p* = .096	*p* = .0005	*p* = 1.0	*p* = .001	*p* = .001
CXCL1	*p* = 1.0	*p* = 1.0	*p* = .037	*p* = 1.0	*p* = .001	*p* = .005
IL-10	*p* = .0005	*p* = .0005	*p* = .0005	*p* = 1.0	*p* = .0005	*p* = .005
IL-16	*p* = .025	*p* = .004	*p* = .005	*p* = 1.0	*p* = .001	*p* = .005
IL-1 beta	*p* = .097	*p* = 1.0	*p* = .027	*p* = 1.0	*p* = .0005	*p* = .001
IL-2	*p* = .179	*p* = .269	*p* = .003	*p* = 1.0	*p* = .260	*p* = .238
IL-4	*p* = .076	*p* = .002	*p* = .0005	*p* = 1.0	*p* = .0005	*p* = .001
IL-6	*p* = .126	*p* = 1.0	*p* = .007	*p* = .427	*p* = .0005	*p* = .001
IL-8	*p* = 1.0	*p* = .059	*p* = .0005	*p* = .035	*p* = .0005	*p* = .005
CXCL10	*p* = 1.0	*p* = 1.0	*p* = .001	*p* = .727	*p* = .0005	*p* = .009
CXCL11	*p* = .037	*p* = .561	*p* = .133	*p* = 1.0	*p* = .0005	*p* = .001
CCL2	*p* = .003	*p* = .006	*p* = .0005	*p* = 1.0	*p* = .001	*p* = .001
CCL8	*p* = 1.0	*p* = 1.0	*p* = .002	*p* = 1.0	*p* = .0005	*p* = .0005
CCL13	*p* = .141	*p* = .001	*p* = .0005	*p* = .619	*p* = .0005	*p* = .005
CCL22	*p* = .138	*p* = .001	*p* = .0005	*p* = .633	*p* = .0005	*p* = .004
MIF	*p* = .167	*p* = .021	*p* = .001	*p* = 1.0	*p* = .002	*p* = .0005
CXCL9	*p* = 1.0	*p* = .568	*p* = .0005	*p* = 1.0	*p* = .002	*p* = .006
CCL3	*p* = 1.0	*p* = 1.0	*p* = .001	*p* = 1.0	*p* = .002	*p* = .022
CCL15	*p* = .016	*p* = .009	*p* = .0005	*p* = 1.0	*p* = .003	*p* = .009
CCL20	*p* = .457	*p* = .281	*p* = .0005	*p* = 1.0	*p* = .009	*p* = .022
CCL19	*p* = .564	*p* = .201	*p* = .0005	*p* = 1.0	*p* = .0005	*p* = .002
CCL23	*p* = .097	*p* = .021	*p* = .0005	*p* = 1.0	*p* = .0005	*p* = .003
CXCL16	*p* = .427	*p* = .007	*p* = .0005	*p* = .574	*p* = .0005	*p* = .011
CXCL12	*p* = 1.0	*p* = 1.0	*p* = .0005	*p* = 1.0	*p* = .001	*p* = .001
CCL25	*p* = .003	*p* = .0005	*p* = 1.0	*p* = 1.0	*p* = .0005	*p* = .0005
TNF-alpha	*p* = .367	*p* = 1.0	*p* = .001	*p* = .407	*p* = .0005	*p* = .0005

The concentration profiles of the 40 cytokines were quantitatively distinct in each of the four groups (controls, PEX, PEXG and PEXL). The levels were notably higher in the PEXL-group than in the other categories (Fig 1).

**Fig 3:**
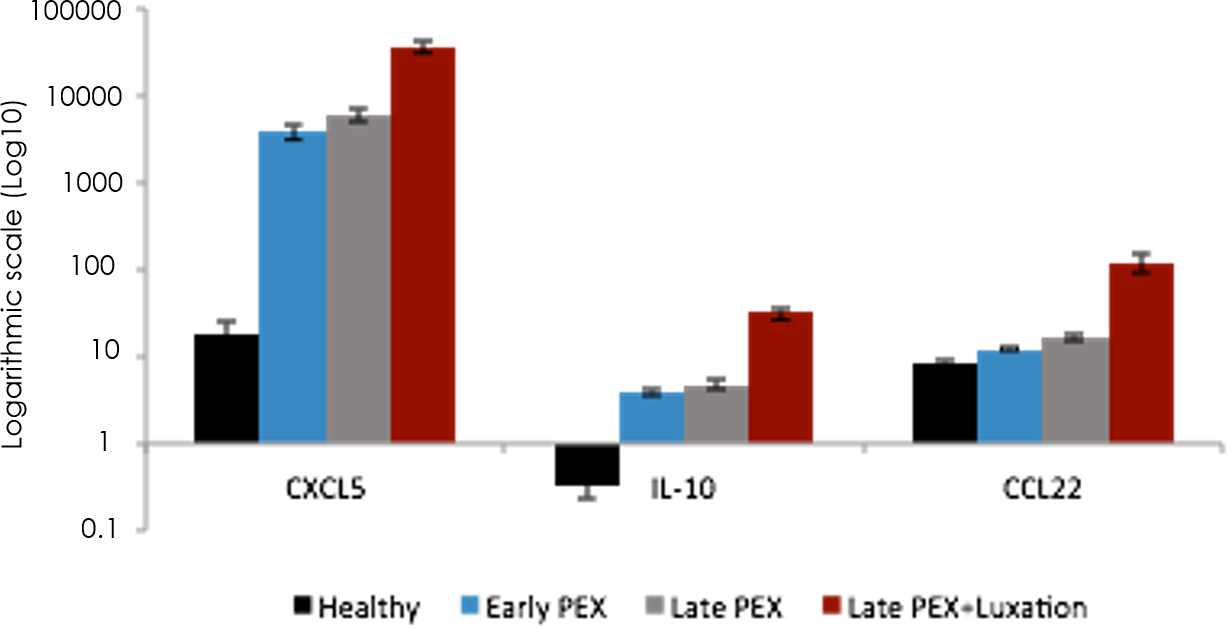
Levels of the cytokines that were most highly expressed in the aqueous humor of eyes with early, late PEX and PEXG with IOL-luxation in comparison to healthy eyes.

CXCL5, IL-10 and CCL2 were detected in each of the three groups of eyes with PEX-syndrome (PEX, PEXG and PEXL, *p* ≤ 0.01 in each category; Fig 3). As was previously reported [16], the levels of TGF-β1 and TGF-β3 were significantly higher in each of these three groups than in the controls (*p* ≤ 0.01 in each category; Fig 3).

CXCL6, CXCL2, CCL1, INF-γ, CCL7 and CCL17 were not detected in the aqueous humor of either the controls or the eyes with PEX-syndrome (PEX, PEXG and PEXL). Hence, these cytokines seem to play no role in healthy and in PEX eyes.

Compared to the controls, CCL25 was down-regulated in PEX-, PEXG- and PEXL-groups (*p*≤0.01; Fig 1, Tables 2 and 3 as well as S3 and S4 Tables). CCL21 was the only cytokine that was down-regulated exclusively in the early PEX-group (*p*≤0.01; Fig 1, Tables 2 and 3).

**Fig 4:**
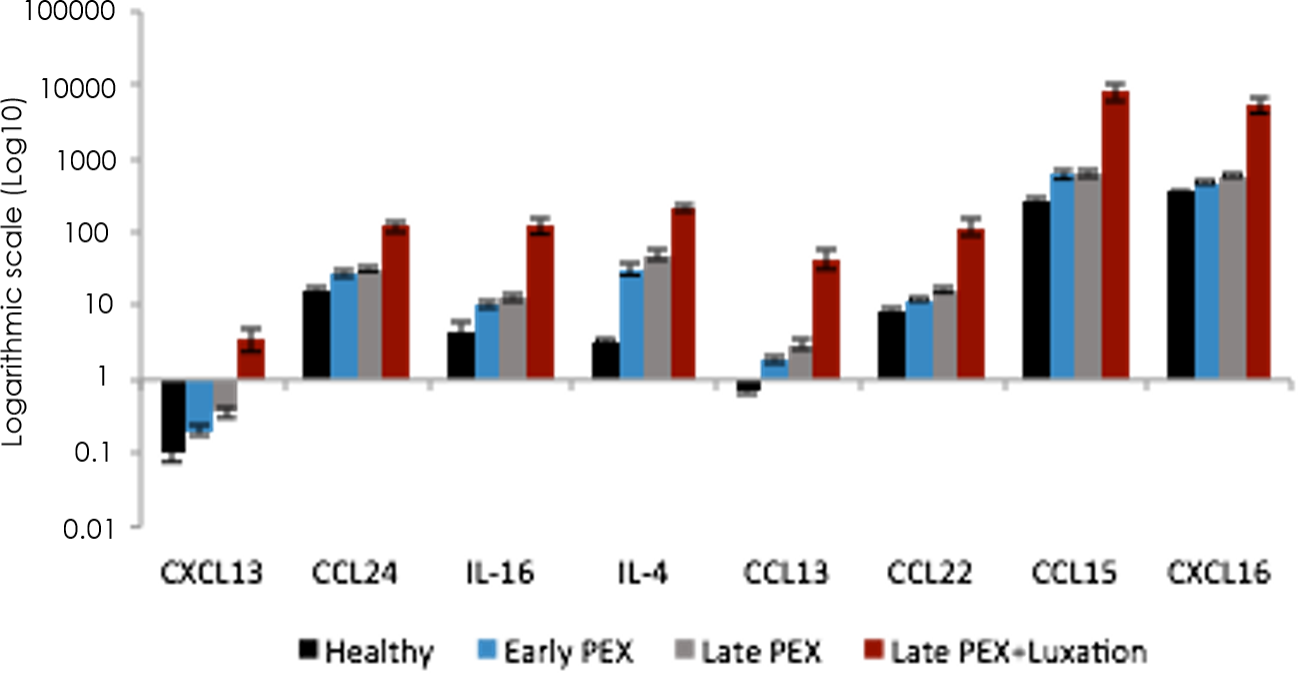
Levels of the cytokines that were most highly expressed in the aqueous humor of eyes with PEXG without and with IOL-luxation when compared to healthy controls.

Compared to the controls, the levels of CXCL13, CCL24, IL-16, IL-4, CCL13, CCL22, CCL15 and CXCL16 were higher in late PEX and late PEX with IOL-luxation, but not in early PEX when compared to healthy controls (*p*≤0.01, each; Fig 4).

**Fig 5:**
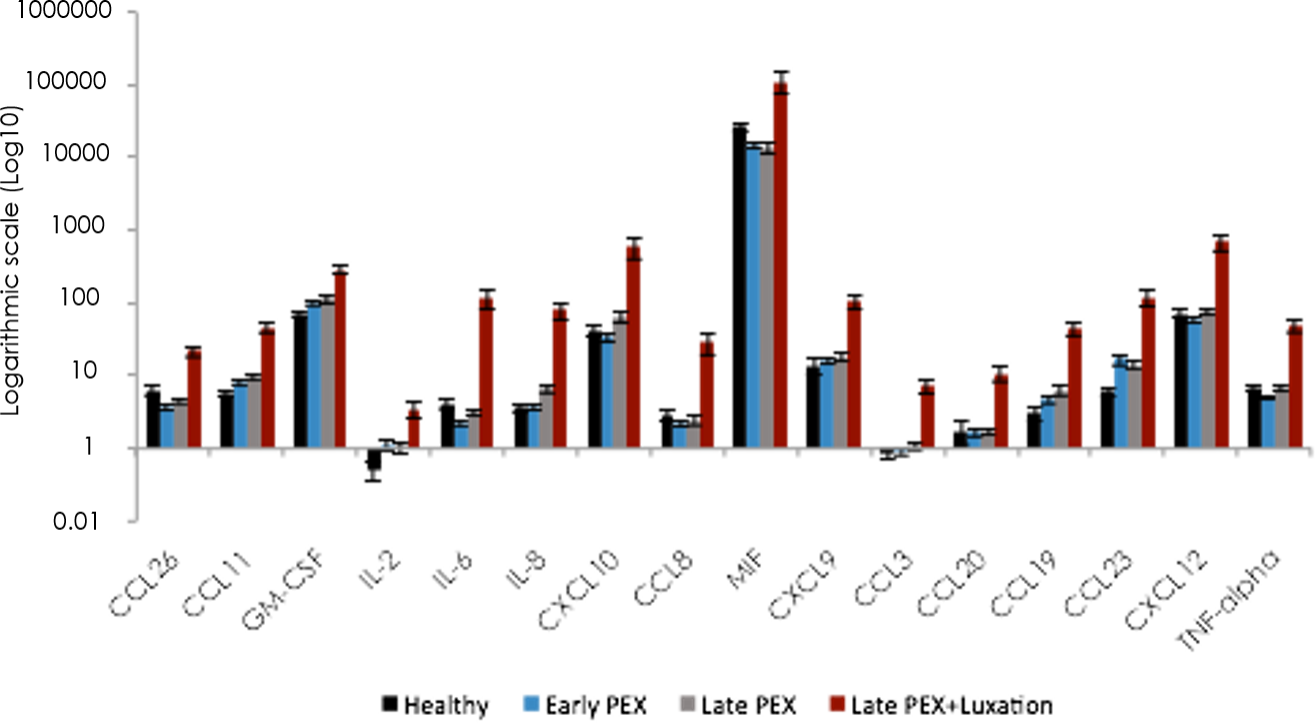
Aqueous levels of the most expressed cytokines in eyes with PEXG with IOL-luxation in comparison to healthy eyes.

In the PEXL-group, but not in any of the other categories, CCL26, CCL11, GM-CSF, IL-2, IL-6, CXCL8, CXCL10, CCL8, MIF, CXCL9, CCL3, CCL20, CCL19, CCL23, CXCL12, and TNF-alpha were up-regulated (p≤0.01, each; Fig 5).

Comparison of PEX and late PEXG without IOL-luxation did not reveal a relevant difference in profiles for the 40 analysed cytokines. However, with the exceptions of CXCL13, CCL27, IL-2, CCL3, CCL20, the detectable 29 cytokines were higher in the PEXL- than in either the PEX or the PEXG-group (Fig 1, Tables 2 and 3).

Presentation of the concentrations of the 40 cytokines in the form of a color-coded heat-map give an overview of the profile differences between the 4 groups. The predominance of the red color (high levels) over green (low levels) reveals that the aqueous humor cytokine concentrations in the PEXL group were higher than in the other groups (Fig 2).

**Fig 6:**
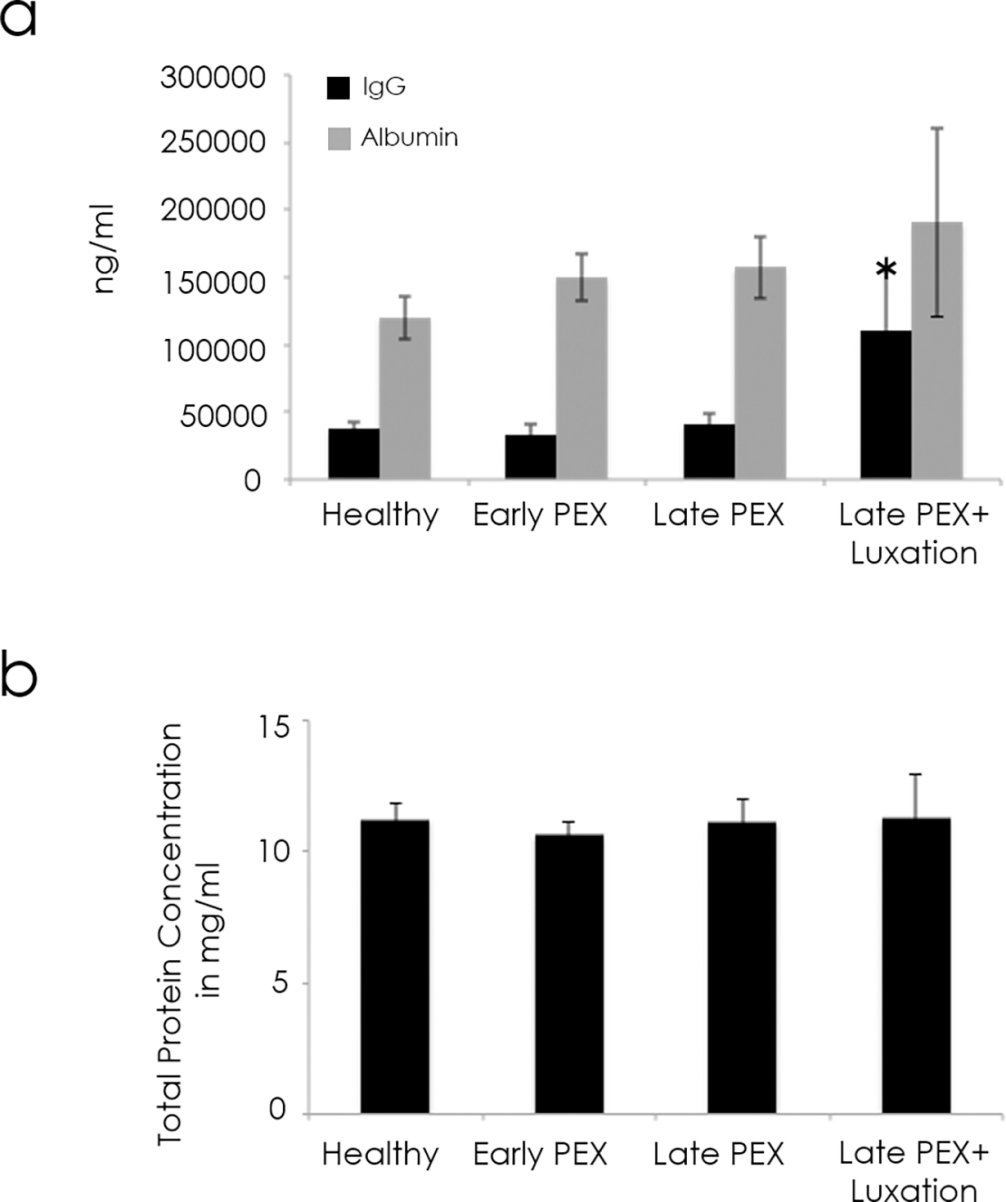
**a.** IgG and Albumin concentrations (ng/ml) in the aqueous humor of all 4 groups. **b.** Total protein concentrations (mg/ml) in the aqueous humor of all 4 groups. *p < 0.05. Error bars represent the standard error of the mean (SEM).

Total protein and albumin concentrations in the aqueous humor of all 4 groups were similar, whereas the IgG levels were increased in the PEXL group (p = 0.02; Fig 6).

## Discussion

Irrespective of the stage of the disease, the aqueous humor cytokine profiles in PEX eyes were similar and comparable to normal controls, with one notable exception: In PEX eyes with luxation of the IOL, the cytokine levels were higher than in those with an intact diaphragm, irrespective of the absence or presence of secondary glaucoma. This is a finding that has not been previously reported and is not readily explained.

To date, elevated levels of only a few selected cytokines, namely, IL-6, IL-8, TNF-alpha and TGf beta-2, have been reported in the aqueous humor of PEX eyes [12–14]. The reported concentrations of these cytokines accord well with our own data. Hence, methodological factors are unlikely to account for the findings in the PEXL- group. Our data indicate that a difference in the level of one particular cytokine between groups may be significant, and yet not meaningful, since the concentrations of many others may as well be abundant. Hence, a comparison of cytokine profiles rather than single cytokines is necessary for an interpretation of environmental changes within a compartment such as the eye.

A possible involvement of systemic inflammation and of endothelial dysfunction in pseudoexfoliation syndrome has been postulated by two independent groups on the basis of serological analyses [17,18]. Our finding that the concentration of IgG, but not total protein or albumin were elevated in the PEXL group affords strong evidence that the upregulation of the cytokines was not accounted for by mechanical damage to the uveovascular barrier and by their consequent leakage from the systemic circulation but by a local progression of the disease towards an inflammatory milieu by the time that the IOL- luxation arises. This situation may exacerbate the already hightened local susceptibility for vascular damage, namely, an increase in the risk for a vasoocclusive disease and an expedition of the progression of glaucomatous optic nerve head damage, in addition to the disease-specific changes in the lamina cribrosa that are associated with a rise in intraocular pressure [3,7]. Not surprisingly in this context, the presence of inflammatory markers, such as IL-6, IL-8, and the vascular endothelial leukocyte-adhesion molecule (ELAM)-1, in the aqueous humor have been linked with the pathogenesis of glaucoma [19–21].

During the early stages of PEX, increases in the concentrations of IL-6 and IL-8 (CXCL-8) in the AH have been demonstrated [12]. Our own data confirmed these findings and revealed, in addition, that the levels of these two cytokines were higher in eyes of the PEXL-group than in those with an early form of the PEX-syndrome. In the aforementioned study [12], cells of the iridal vascular endothelium and those of the non-pigmented ciliary epithelium were demonstrated to be a source of IL-6, but not IL-8. Hypoxia and oxidative stress were proposed to induce, at an early stage of PEX-syndrome, a state of–temporally restricted, subclinical inflammation, as evidenced by cytokine upregulation, which resulted in the production of TGF beta-1 and of elastic fiber proteins [12]. Our own data revealed no increase in the concentration of the cytokines in the aqueous humor with the progression of the early form of PEX-syndrome to an advanced, glaucomatous one. Our finding that the cytokine levels were higher in the PEXL- than in the PEX-group may either be indicative of oxidative stress-induced inflammatory changes also at this stage of the disease, or, alternatively, reflect mechanical damage to the diaphragm that was induced by the IOL itself or by vitreal prolapse. However, in the absence of a breakdown of the uveovascular barrier, a situation such as the latter is less likely to be of pathophysiological relevance.

The aqueous humor levels of CXCL13, CCL24, IL-16, IL-4, CCL13, CCL22, CCL15 and CXCL16 were higher in the PEXG—and the PEXL-groups than in the control and the early PEX eyes. All of these cytokines share in common chemoattractant properties and, with the exception of IL-4, they are pro-inflammatory. Since IL-4 can induce CCL2 (MCP-1) it contributes indirectly to inflammatory processes [22]. The question arises as to whether these eight cytokines could serve as markers for PEX progression. However, the pleiotropic characteristics of cytokines renders an exact assignment difficult. The presence of various chemoattractants and mediators in the aqueous humor of eyes with pseudoexfoliation implicates an involvement of multifunctional cells which may orchestrate or are induced in response to the degenerative tissue changes. Principally, the involvement of neutrophils, macrophages and dendritic cells, but also basophiles, eosinophils and mast cells might be conceivable and from the adaptive system lymphocytes, in particular T cells. Since these cells are clinically not normally detectable in the aqueous humor, we assume that the aqueous cytokines are derived from uveal tissue. According to immunohistochemical data in this disease, these are primarily generated in response to tissue hypoxia and the accumulation of free oxygen radicals probably merely by uveal pigment epithelial cells [12, 23, 24], nevertheless evidence regarding this point is scarce since other cells have not been excluded. Co-factors of the PEX-specific fibrotic matrix process include elevated levels of fibrogenic growth factors, TGF-beta1-3, reduced activity of proteolytic enzymes, subtle inflammatory processes and various external stress factors, such as oxidative stress ^3^. Previously PEX has been linked solely to TGF-beta 1 [25], later also to TGF-beta 2 [12]. Recently, a negative correlation has been demonstrated to exist between LOX activity in the aqueous humor and the concentrations therein of TGF-beta1 and TGF-beta2, thereby confirming exsisting data [26]. Takai et al. showed a positive correlation of TGF-beta 1, IL-8 and SAA with IOP in open angle and exfoliation glaucoma [27], whereas our results display a positive correlation for CXCL13, CCL8 and CCL3 in the PEXG-group, but no other correlation in any of the four groups. Since the observed correlations do not follow any systematic pattern, we believe that these may well be random observations based on the limited number of patients.

We are obviously still far from pinpointing the impact of single factors on the environmental changes that occur in the anterior segment of eyes with PEX-syndrome [10,12]. Since the disease develops in aging tissues and shares many features in common with physiological, age-related, low-grade inflammation (inflammaging), a distinction between these two states cannot be made. They may indeed be one and the same process. Changes in the levels not only of single chemo- and cytokines, but the whole cytokine profile are orchestrating the response to an altered LOXL1 activity, and these changes seem to follow or lead the progression of this disease.

## Supporting information

S1 Table Correlations of the 40 cytokines with age. The correlation coefficient (ρ) and *p-*values for each cytokine and for each group are calculated by Spearman’s correlation test. * p<0.05, ** p<0.01.
(DOCX)

S2 Table Correlations of the 40 cytokines with IOP. The correlation coefficient (ρ) and *P* values for each cytokine and for each group are calculated by Spearman’s correlation test. * p<0.05, ** p<0.01.
(DOCX)

S3 Table Complete information appertaining to the 40 cytokines and chemokines whose levels in the aqueous humor were quantified using the Bio-Plex multiplex beads system. (DOCX)

S4 Table Complete information (*p*-values) appertaining to the 40 cytokines and chemokines whose levels in the aqueous humor were quantified using the Bio-Plex multiplex beads system. (DOCX)
