# Supplementary material for: Comparison of cytokine profiles in the aqueous humor of eyes with pseudoexfoliation syndrome and glaucoma
Source: PLoS One. 2017 Aug 10;12(8):e0182571. doi: 10.1371/journal.pone.0182571 (PMC5552219; doi:10.1371/journal.pone.0182571)
Supplement: S1 Table — The correlation coefficient (ρ) and p-values for each cytokine and for each group are calculated by Spearman’s correlation test. * p<0.05, ** p<0.01. (DOCX) [file pone.0182571.s001.docx]

|  | Age (years) | | | | | | | |
| --- | --- | --- | --- | --- | --- | --- | --- | --- |
|  | Healthy | | Early PEX | | Late PEX | | Late PEX+ luxation | |
| Cytokine | ρ | *P* value | ρ | *P* value | ρ | *P* value | ρ | *P* value |
| CCL21 | 0.38 | 0.10 | 0.10 | 0.62 | 0.16 | 0.41 | -0.18 | 0.63 |
| CXCL13 | 0.40 | 0.10 | 0.36 | 0.07 | 0.28 | 0.17 | 0.17 | 0.69 |
| CCL27 | n.a. | n.a. | 0.33 | 0.07 | 0.23 | 0.91 | -0.78 | 0.07 |
| CXCL5 | 0.31 | 0.25 | -0.30 | 0.11 | 0.29 | 0.13 | 0.04 | 0.91 |
| CCL24 | -0.08 | 0.77 | -0.17 | 0.38 | 0.34 | 0.08 | 0.18 | 0.64 |
| CCL26 | 0.38 | 0.11 | 0.08 | 0.67 | 0.38 | **0.045*** | -0.09 | 0.80 |
| CCL11 | 0.13 | 0.60 | -0.11 | 0.55 | 0.26 | 0.21 | -0.23 | 0.55 |
| CX3CL1 | 0.17 | 0.47 | 0.18 | 0.35 | 0.24 | 0.23 | 0.32 | 0.37 |
| CXCL6 | Not detectable | | | | | | | |
| GM-CSF | -0.05 | 0.83 | -0.29 | 0.12 | 0.01 | 0.97 | -0.56 | 0.09 |
| CXCL1 | 0.48 | **0.032*** | 0.35 | 0.06 | 0.21 | 0.29 | -0.06 | 0.87 |
| CXCL2 | Not detectable | | | | | | | |
| CCL1 | Not detectable | | | | | | | |
| IFN- γ | Not detectable | | | | | | | |
| IL-10 | 0.08 | 0.78 | 0.19 | 0.32 | 0.27 | 0.18 | -0.01 | 0.99 |
| IL-16 | 0.12 | 0.62 | 0.03 | 0.85 | 0.46 | **0.014*** | -0.49 | 0.15 |
| IL-1 Beta | 0.31 | 0.18 | 0.09 | 0.65 | 0.40 | **0.035*** | -0.29 | 0.44 |
| IL-2 | 0.05 | 0.83 | -0.26 | 0.14 | 0.32 | 0.10 | -0.28 | 0.43 |
| IL-4 | 0.14 | 0.56 | -0.30 | 0.11 | 0.20 | 0.31 | -0.44 | 0.20 |
| IL-6 | -0.02 | 0.94 | -0.01 | 0.96 | -0.02 | 0.92 | -0.45 | 0.26 |
| IL-8 | 0.40 | 0.09 | 0.46 | **0.011*** | 0.02 | 0.92 | -0.03 | 0.93 |
| CXCL10 | 0.39 | 0.09 | 0.22 | 0.26 | 0.33 | 0.11 | -0.12 | 0.75 |
| CXCL11 | 0.36 | 0.14 | 0.22 | 0.22 | 0.52 | **0.004**** | -0.20 | 0.58 |
| CCL2 | 0.36 | 0.12 | 0.48 | **0.008**** | 0.23 | 0.27 | -0.02 | 0.96 |
| CCL8 | 0.43 | 0.06 | 0.06 | 0.73 | -0.10 | 0.63 | -0.06 | 0.88 |
| CCL7 | Not detectable | | | | | | | |
| CCL13 | 0.34 | 0.14 | -0.08 | 0.69 | 0.14 | 0.48 | -0.37 | 0.29 |
| CCL22 | 0.28 | 0.26 | -0.11 | 0.55 | 0.27 | 0.16 | -0.15 | 0.68 |
| MIF | 0.35 | 0.17 | 0.12 | 0.55 | 0.06 | 0.76 | -0.27 | 0.49 |
| CXCL9 | 0.51 | **0.022*** | 0.13 | 0.49 | 0.56 | **0.003**** | -0.13 | 0.73 |
| CCL3 | 0.41 | 0.07 | 0.26 | 0.17 | -0.15 | 0.44 | -0.14 | 0.70 |
| CCL15 | 0.47 | **0.04*** | 0.13 | 0.50 | 0.42 | **0.028*** | -0.10 | 0.78 |
| CCL20 | 0.12 | 0.61 | 0.32 | 0.09 | 0.06 | 0.76 | 0.03 | 0.93 |
| CCL19 | -0.08 | 0.75 | -0.19 | 0.32 | 0.21 | 0.30 | -0.05 | 0.91 |
| CCL23 | -0.43 | 0.08 | -0.02 | 0.94 | 0.30 | 0.14 | 0.18 | 0.63 |
| CXCL16 | 0.44 | 0.06 | 0.46 | **0.01**** | 0.13 | 0.51 | -0.25 | 0.49 |
| CXCL12 | -0.12 | 0.63 | 0.09 | 0.63 | 0.01 | 0.97 | 0.08 | 0.83 |
| CCL17 | Not detectable | | | | | | | |
| CCL25 | 0.46 | **0.046*** | 0.13 | 0.46 | 0.29 | 0.15 | -0.57 | 0.11 |
| TNF-apha | 0.39 | 0.09 | 0.06 | 0.76 | 0.40 | **0.04*** | -0.16 | 0.66 |

The correlation coefficient (ρ) and *p-*values for each cytokine and for each group are calculated by Spearman’s correlation test. * p<0.05, ** p<0.01.
