# Supplementary material for: Comparison of cytokine profiles in the aqueous humor of eyes with pseudoexfoliation syndrome and glaucoma
Source: PLoS One. 2017 Aug 10;12(8):e0182571. doi: 10.1371/journal.pone.0182571 (PMC5552219; doi:10.1371/journal.pone.0182571)
Supplement: S3 Table — (DOCX) [file pone.0182571.s003.docx]

| Cytokine | Healthy | | | | | Early PEX | | | | | Late PEX | | | | | late PEX + luxation | | | | |
| --- | --- | --- | --- | --- | --- | --- | --- | --- | --- | --- | --- | --- | --- | --- | --- | --- | --- | --- | --- | --- |
|  | Mean  (pg/ml) | SD | Range | | N | Mean  (pg/ml) | SD | Range | | N | Mean  (pg/ml) | SD | Range | | N | Mean  (pg/ml) | SD | Range | | N |
|  |  |  | Min. | Max. |  |  |  | Min. | Max. |  |  |  | Min. | Max. |  |  |  | Min. | Max. |  |
| CCL21 | 1585.9 | 1007.4 | 708.6 | 4265.0 | 19 | 787.8 | 367.3 | 307.2 | 1640.8 | 30 | 1013.5 | 469.0 | 362.2 | 2210.6 | 27 | 5292.0 | 3522.7 | 1469.4 | 11161.2 | 10 |
| CXCL13 | 0.1 | 0.1 | 0.0 | 0.3 | 18 | 0.2 | 0.2 | 0.0 | 0.6 | 27 | 0.4 | 0.3 | 0.0 | 1.1 | 26 | 3.7 | 3.8 | 0.0 | 11.4 | 8 |
| CCL27 | 0.0 | 0.0 | 0.0 | 0.0 | 17 | 1.0 | 1.6 | 0.0 | 5.5 | 31 | 0.6 | 1.6 | 0.0 | 5.5 | 25 | 2.2 | 5.1 | 0.0 | 12.6 | 6 |
| CXCL5 | 18.2 | 27.0 | 11.4 | 119.6 | 16 | 3905.5 | 4318.2 | 11.4 | 11000.6 | 30 | 6130.1 | 6044.9 | 11.4 | 21913.3 | 28 | 36834.2 | 18300.0 | 9619.1 | 63684.46 | 10 |
| CCL24 | 16.8 | 6.7 | 9.0 | 34.1 | 17 | 27.2 | 12.2 | 12.6 | 62.8 | 30 | 32.4 | 16.8 | 12.2 | 69.8 | 28 | 120.2 | 70.3 | 35.5 | 256.5 | 9 |
| CCL26 | 6.4 | 4.8 | 2.4 | 19.9 | 19 | 3.7 | 1.6 | 0.3 | 7.4 | 31 | 4.5 | 1.8 | 2.2 | 8.1 | 28 | 21.8 | 11.7 | 7.8 | 37.3 | 10 |
| CCL11 | 5.7 | 2.3 | 2.5 | 9.8 | 20 | 8.2 | 4.2 | 3.1 | 15.7 | 30 | 9.8 | 5.0 | 1.7 | 21.4 | 26 | 45.1 | 18.7 | 13.0 | 62.5 | 9 |
| CX3CL1 | 47.2 | 24.5 | 23.1 | 97.8 | 20 | 33.2 | 13.1 | 14.2 | 67.2 | 30 | 32.0 | 12.0 | 12.0 | 57.9 | 27 | 126.1 | 91.4 | 21.1 | 295.5 | 10 |
| CXCL6 | not detectable | | | | | | | | | | | | | | | | | | | |
| GM-CSF | 69.0 | 20.8 | 30.6 | 116.3 | 20 | 98.0 | 33.1 | 52.3 | 182.4 | 30 | 111.7 | 71.5 | 29.5 | 302.3 | 28 | 280.3 | 107.2 | 102.4 | 407.6 | 10 |
| CXCL1 | 36.7 | 17.1 | 4.6 | 65.2 | 20 | 29.8 | 17.1 | 4.6 | 58.0 | 30 | 32.8 | 18.7 | 4.6 | 72.9 | 27 | 168.8 | 93.6 | 21.6 | 284.1 | 10 |
| CXCL2 | not detectable | | | | | | | | | | | | | | | | | | | |
| CCL1 | not detectable | | | | | | | | | | | | | | | | | | | |
| IFN- γ | not detectable | | | | | | | | | | | | | | | | | | | |
| IL-10 | 0.3 | 0.4 | 0.2 | 1.3 | 17 | 3.8 | 1.5 | 1.0 | 6.9 | 30 | 4.7 | 2.7 | 0.2 | 11.2 | 27 | 31.6 | 16.4 | 6.9 | 51.2 | 10 |
| IL-16 | 4.7 | 5.6 | 0.7 | 21.6 | 20 | 10.4 | 5.9 | 0.7 | 22.7 | 32 | 13.1 | 9.4 | 0.7 | 35.2 | 28 | 123.6 | 91.5 | 13.8 | 275.8 | 10 |
| IL-1 beta | 0.6 | 0.4 | 0.0 | 1.3 | 20 | 0.4 | 0.3 | 0.0 | 1.0 | 30 | 0.5 | 0.4 | 0.0 | 1.4 | 28 | 2.3 | 1.4 | 0.6 | 4.7 | 9 |
| IL-2 | 0.5 | 0.6 | 0.1 | 2.1 | 20 | 1.1 | 1.2 | 0.1 | 3.8 | 33 | 1.0 | 1.0 | 0.1 | 3.1 | 28 | 3.5 | 2.7 | 0.1 | 8.4 | 10 |
| IL-4 | 3.4 | 0.9 | 3.2 | 7.0 | 20 | 32.0 | 31.7 | 3.2 | 81.6 | 30 | 48.6 | 40.6 | 3.2 | 152.6 | 28 | 212.5 | 73.3 | 68.5 | 282.4 | 10 |

| Cytokine | Healthy | | | | | Early PEX | | | | | Late PEX | | | | | late PEX + luxation | | | | |
| --- | --- | --- | --- | --- | --- | --- | --- | --- | --- | --- | --- | --- | --- | --- | --- | --- | --- | --- | --- | --- |
|  | Mean  (pg/ml) | SD | Range | | N | Mean  (pg/ml) | SD | Range | | N | Mean  (pg/ml) | SD | Range | | N | Mean  (pg/ml) | SD | Range | | N |
|  |  |  | Min. | Max. |  |  |  | Min. | Max. |  |  |  | Min. | Max. |  |  |  | Min. | Max. |  |
| IL-6 | 4.1 | 2.7 | 1.3 | 10.9 | 19 | 2.2 | 0.8 | 0.6 | 3.4 | 27 | 3.1 | 1.5 | 1.2 | 6.6 | 23 | 118.6 | 104.9 | 6.5 | 284.3 | 8 |
| IL-8 | 3.7 | 2.1 | 0.8 | 8.9 | 19 | 3.7 | 1.4 | 1.4 | 6.8 | 30 | 6.5 | 4.1 | 1.0 | 17.0 | 29 | 79.8 | 66.4 | 8.2 | 169.4 | 10 |
| CXCL10 | 42.0 | 32.1 | 2.6 | 113.4 | 20 | 33.4 | 23.2 | 5.5 | 99.5 | 28 | 64.2 | 61.1 | 8.2 | 236.3 | 25 | 590.8 | 609.7 | 21.8 | 1918.4 | 10 |
| CXCL11 | 1.7 | 1.3 | 0.3 | 4.4 | 18 | 0.7 | 0.4 | 0.1 | 1.7 | 33 | 0.9 | 0.5 | 0.1 | 2.0 | 28 | 4.4 | 2.4 | 0.8 | 7.4 | 10 |
| CCL2 | 244.5 | 68.2 | 131.4 | 351.5 | 20 | 368.1 | 125.5 | 174.0 | 702.4 | 30 | 361.6 | 117.1 | 185.8 | 662.0 | 26 | 2296.5 | 1696.5 | 528.4 | 5493.1 | 10 |
| CCL8 | 2.8 | 2.1 | 0.2 | 7.4 | 20 | 2.2 | 1.1 | 0.6 | 5.0 | 31 | 2.4 | 1.8 | 0.4 | 7.5 | 27 | 28.9 | 30.6 | 1.8 | 90.5 | 10 |
| CCL7 |  |  |  |  |  |  |  |  |  |  |  |  |  |  |  |  |  |  |  |  |
| CCL13 | 0.7 | 0.5 | 0.0 | 1.8 | 20 | 1.8 | 1.2 | 0.4 | 3.8 | 30 | 3.0 | 2.2 | 0.3 | 8.9 | 27 | 43.9 | 39.1 | 4.1 | 120.1 | 10 |
| CCL22 | 8.2 | 3.9 | 2.3 | 15.6 | 19 | 12.1 | 4.3 | 5.6 | 19.8 | 30 | 16.8 | 8.9 | 6.7 | 37.2 | 28 | 118.8 | 90.6 | 28.1 | 304.1 | 10 |
| MIF | 26343.3 | 14414.9 | 2386.7 | 51732.4 | 17 | 14766.7 | 9515.2 | 1471.4 | 36441.7 | 30 | 13323.8 | 13160.4 | 1994.9 | 42847.2 | 28 | 108694.3 | 106911.8 | 4131.6 | 277153.8 | 9 |
| CXCL9 | 13.5 | 15.3 | 1.3 | 45.2 | 20 | 16.1 | 10.9 | 2.5 | 42.7 | 31 | 17.8 | 11.8 | 1.3 | 44.0 | 26 | 102.6 | 63.8 | 16.3 | 213.1 | 9 |
| CCL3 | 0.8 | 0.7 | 0.1 | 2.0 | 20 | 0.9 | 0.5 | 0.1 | 2.1 | 30 | 1.1 | 0.7 | 0.1 | 2.8 | 29 | 7.2 | 5.3 | 0.1 | 15.9 | 10 |
| CCL15 | 266.5 | 144.2 | 93.9 | 505.2 | 19 | 611.1 | 448.6 | 119.7 | 1857.6 | 32 | 611.5 | 373.2 | 174.5 | 1456.9 | 27 | 8211.6 | 7528.7 | 318.8 | 21719.8 | 10 |
| CCL20 | 1.7 | 2.6 | 0.1 | 7.2 | 20 | 1.7 | 1.2 | 0.1 | 4.4 | 30 | 1.7 | 0.9 | 0.1 | 4.3 | 26 | 10.7 | 7.3 | 0.1 | 21.6 | 9 |
| CCL19 | 3.1 | 3.3 | 0.5 | 11.2 | 20 | 4.6 | 2.6 | 0.5 | 9.9 | 29 | 6.4 | 5.5 | 0.5 | 24.2 | 27 | 44.9 | 27.1 | 10.4 | 84.6 | 8 |
| CCL23 | 6.0 | 3.9 | 0.8 | 14.4 | 18 | 16.8 | 16.8 | 0.8 | 60.1 | 32 | 14.0 | 7.7 | 0.8 | 31.6 | 26 | 118.8 | 95.7 | 19.5 | 276.6 | 10 |
| CXCL16 | 362.2 | 94.0 | 228.1 | 590.3 | 19 | 471.4 | 166.3 | 233.5 | 801.1 | 30 | 586.9 | 232.9 | 216.2 | 1210.4 | 27 | 5453.5 | 4162.0 | 498.8 | 13843.8 | 10 |
| CXCL12 | 71.2 | 39.5 | 24.2 | 159.9 | 20 | 59.7 | 34.5 | 22.2 | 157.6 | 32 | 76.5 | 48.0 | 24.1 | 223.3 | 27 | 690.8 | 563.9 | 56.2 | 1545.7 | 10 |
| CCL17 | not detectable | | | | | | | | | | | | | | | | | | | |
| CCL25 | 118.2 | 74.5 | 37.1 | 283.0 | 19 | 51.1 | 27.1 | 16.5 | 129.6 | 32 | 42.6 | 25.0 | 17.9 | 103.2 | 26 | 289.2 | 179.3 | 33.8 | 602.4 | 9 |
| TNF-alpha | 6.8 | 3.5 | 1.9 | 14.6 | 20 | 5.1 | 1.8 | 1.8 | 9.2 | 30 | 6.6 | 3.2 | 1.2 | 16.9 | 27 | 47.8 | 26.9 | 9.5 | 79.9 | 10 |
