# Supplementary material for: Comparison of cytokine profiles in the aqueous humor of eyes with pseudoexfoliation syndrome and glaucoma
Source: PLoS One. 2017 Aug 10;12(8):e0182571. doi: 10.1371/journal.pone.0182571 (PMC5552219; doi:10.1371/journal.pone.0182571)
Supplement: S4 Table — (DOCX) [file pone.0182571.s004.docx]

|  | **Kruskal-Wallis H-Test** | **Kruskal-Wallis H-Test** | **Kruskal-Wallis H-Test** | **Kruskal-Wallis H-Test** | **Kruskal-Wallis H-Test** | **Kruskal-Wallis H-Test** |
| --- | --- | --- | --- | --- | --- | --- |
| **Cytokine** | **Healthy** | **Healthy** | **Healthy** | **Early PEX** | **Early PEX** | **Late PEX** |
|  | **Early PEX** | **Late PEX** | **Late PEX+ luxation** | **Late PEX** | **Late PEX+ luxation** | **Late PEX+ luxation** |
| CCL21 | *p*=0.006 | *p*=.382 | *p*=.062 | *p=*.723 | *p=*.0005 | *p=*.0005 |
| CXCL13 | *p*=.39 | *p*=.008 | *p*=.0005 | *p=*.767 | *p=*.005 | *p=*.139 |
| CCL27 | *p*=.037 | *p*=.816 | *p*=.550 | *p=*1.0 | *p=*1.0 | *p=*1.0 |
| CXCL5 | *p*=.003 | *p*= .0005 | *p*= .0005 | *p=*1.0 | *p=*.0005 | *p=*.002 |
| CCL24 | *p*= .039 | *p*= .004 | *p*= .0005 | *p=*1.0 | *p=*.001 | *p=*.005 |
| CCL26 | *p*= .224 | *p*= 1.0 | *p*= .005 | *p*= 1.0 | *p=*.0005 | *p=*.0005 |
| CCL11 | *p*= .787 | *p*= .080 | *p*= .0005 | *p=1.0* | *p=*.0005 | *p=.003* |
| CX3CL1 | *p*= .610 | *p*= .343 | *p*= .532 | *p*= 1.0 | *p*= .012 | *p*= .006 |
| CXCL6 | not detectable | | | | | |
| GM-CSF | *p*= .063 | *p*= .096 | *p*= .0005 | *p*= 1.0 | *p*= .001 | *p*= .001 |
| CXCL1 | *p*= 1.0 | *p*= 1.0 | *p*= .037 | *p*= 1.0 | *p*= .001 | *p*= .005 |
| CXCL2 | not detectable | | | | | |
| CCL1 | not detectable | | | | | |
| IFN- γ | not detectable | | | | | |
| IL-10 | *p*= .0005 | *p*= .0005 | *p*= .0005 | *p*= 1.0 | *p*= .0005 | *p*= .005 |
| IL-16 | *p*= .025 | *p*= .004 | *p*= .005 | *p*= 1.0 | *p*= .001 | *p*= .005 |
| IL-1 beta | *p*= .097 | *p*= 1.0 | *p*= .027 | *p*= 1.0 | *p*= .0005 | *p*= .001 |
| IL-2 | *p*= .179 | *p*= .269 | *p*= .003 | *p*= 1.0 | *p*= .260 | *p*= .238 |
| IL-4 | *p*= .076 | *p*= .002 | *p*= .0005 | *p*= 1.0 | *p*= .0005 | *p*= .001 |
| IL-6 | *p*= .126 | *p*= 1.0 | *p*= .007 | *p*= .427 | *p*= .0005 | *p*= .001 |
| IL-8 | *p*= 1.0 | *p*= .059 | *p*= .0005 | *p*= .035 | *p*= .0005 | *p*= .005 |
| CXCL10 | *p*= 1.0 | *p*= 1.0 | *p*= .001 | *p*= .727 | *p*= .0005 | *p*= .009 |
| CXCL11 | *p*= .037 | *p*= .561 | *p*= .133 | *p*= 1.0 | *p*= .0005 | *p*= .001 |
| CCL2 | *p*= .003 | *p*= .006 | *p*= .0005 | *p*= 1.0 | *p*= .001 | *p*= .001 |
| CCL8 | *p*= 1.0 | *p*= 1.0 | *p*= .002 | *p*= 1.0 | *p*= .0005 | *p*= .0005 |
| CCL7 | Not detectable | | | | | |
| CCL13 | *p*= .141 | *p*= .001 | *p*= .0005 | *p*= .619 | *p*= .0005 | *p*= .005 |
| CCL22 | *p*= .138 | *p*= .001 | *p*= .0005 | *p*= .633 | *p*= .0005 | *p*= .004 |
| MIF | *p*= .167 | *p*= .021 | *p*= .001 | *p*= 1.0 | *p*= .002 | *p*= .0005 |
| CXCL9 | *p*= 1.0 | *p*= .568 | *p*= .0005 | *p*= 1.0 | *p*= .002 | *p*= .006 |
| CCL3 | *p*= 1.0 | *p*= 1.0 | *p*= .001 | *p*= 1.0 | *p*= .002 | *p*= .022 |
| CCL15 | *p*= .016 | *p*= .009 | *p*= .0005 | *p*= 1.0 | *p*= .003 | *p*= .009 |
| CCL20 | *p*= .457 | *p*= .281 | *p*= .0005 | *p*= 1.0 | *p*= .009 | *p*= .022 |
| CCL19 | *p*= .564 | *p*= .201 | *p*= .0005 | *p*= 1.0 | *p*= .0005 | *p*= .002 |
| CCL23 | *p*= .097 | *p*= .021 | *p*= .0005 | *p*= 1.0 | *p*= .0005 | *p*= .003 |
| CXCL16 | *p*= .427 | *p*= .007 | *p*= .0005 | *p*= .574 | *p*= .0005 | *p*= .011 |
| CXCL12 | *p*= 1.0 | *p*= 1.0 | *p*= .0005 | *p*= 1.0 | *p*= .001 | *p*= .001 |
| CCL17 | Not detectable | | | | | |
| CCL25 | *p*= .003 | *p*= .0005 | *p*= 1.0 | *p*= 1.0 | *p*= .0005 | *p*= .0005 |
| TNF-alpha | *p*= .367 | *p*= 1.0 | *p*= .001 | *p*= .407 | *p*= .0005 | *p*= .0005 |
